# Isolation of *Brucella inopinata* from a White’s tree frog (*Litoria caerulea*): pose exotic frogs a potential risk to human health?

**DOI:** 10.3389/fmicb.2023.1173252

**Published:** 2023-06-08

**Authors:** Holger C. Scholz, Kim O. Heckers, Sandra Appelt, Dorothee Geier-Dömling, Patrick Schlegel, Alice R. Wattam

**Affiliations:** ^1^Department of Bacteriology and Toxinology, Bundeswehr Institute of Microbiology, Munich, Germany; ^2^LABOklin GmbH and Co KG, Labor für klinische Diagnostik, Bad Kissingen, Germany; ^3^Kleintierpraxis Dr. med vet. Patrick Schlegel, Sargans, Switzerland; ^4^Biocomplexity Institute, University of Virginia, Charlottesville, VA, United States

**Keywords:** *Brucella inopinata*, atypical *Brucella*, exotic frogs, public health, genomics

## Abstract

**Introduction:**

Cold-blooded hosts, particularly exotic frogs, have become a newly recognized reservoir for atypical *Brucella* species and strains worldwide, but their pathogenicity to humans remains largely unknown. Here we report the isolation and molecular characterization of a *B. inopinata* strain (FO700662) cultured from clinical samples taken from a captive diseased White’s Tree Frog (*Litoria caerulea*) in Switzerland. The isolation of *B. inopinata* from a frog along with other reports of human infection by atypical Brucella raises the question of whether atypical Brucella could pose a risk to human health and deserves further attention.

**Methods:**

The investigations included histopathological analysis of the frog, bacterial culture and in-depth molecular characterization of strain FO700662 based on genome sequencing data.

**Results and Discussion:**

Originally identified as *Ochrobactrum* based on its rapid growth and biochemical profile, strain FO700622 was positive for the *Brucella-* specific markers *bcsp31* and *IS711*. It showed the specific banding pattern of *B. inopinata* in conventional Bruce-ladder multiplex PCR and also had identical 16S rRNA and recA gene sequences as *B. inopinata*. Subsequent genome sequencing followed by core genome-based MLST (cgMLST) analysis using 2704 targets (74% of the total chromosome) revealed only 173 allelic differences compared to the type strain of *B. inopinata* BO1^T^, while previously considered the closest related strain BO2 differed in 2046 alleles. The overall average nucleotide identity (ANI) between the type strain BO1^T^ and FO700622 was 99,89%, confirming that both strains were almost identical. In *silico* MLST-21 and MLVA-16 also identified strain FO700662 as *B. inopinata*. The nucleotide and amino acid-based phylogenetic reconstruction and comparative genome analysis again placed the isolate together with *B. inopinata* with 100% support. In conclusion, our data unequivocally classified strain FO700622, isolated from an exotic frog, as belonging to *B. inopinata*.

## Introduction

Many members of the genus *Brucella* are important zoonotic pathogens that can infect various animal species and humans (Godfroid et al., 2005; Seleem et al., 2010). The resulting disease, brucellosis, is one of the most common bacterial zoonoses worldwide with an estimated incidence of 500,000 human cases annually (Pappas et al., 2006b). The majority of human cases are caused by *B. melitensis*, followed by *B. abortus* and *B. suis*, with other species only rarely causing disease in man. Because of the low infection dose and possible transmission as an aerosol, *B. melitensis* and most other *Brucella* species, including *B. inopinata*, are classified as a risk level 3 (category A) pathogens (Pappas et al., 2006a).

For decades, the genus *Brucella* consisted exclusively of the classical *Brucella* species (*B. melitensis*, *B. abortus*, *B. suis*, *B. canis*, *B. ovis*, and *B. neotomae*). However, in recent years, the genus has expanded rapidly from terrestrial and marine mammals to fish, amphibians, and even reptiles (Godfroid et al., 2005; Foster et al., 2007; De et al., 2008; Scholz et al., 2008c, 2016a,b; Whatmore et al., 2014; Muhldorfer et al., 2017; Eisenberg et al., 2020).

Many of these novel *Brucella* isolates have either atypical biochemical or molecular characteristics compared to the group of closely related classical *Brucella* species (Scholz and Vergnaud, 2013). Consequently, the genus is now divided into the classical species (also referred to as “core *Brucella*”), including strains of marine mammals (*B. ceti* and *B. pinnipedialis*), and the genomes composed of genetically and biochemically more diverse species and isolates (Scholz et al., 2008a; Whatmore, 2009; Whatmore et al., 2016; Ashford et al., 2020). While the pathogenicity of classical species to humans is well documented, these data are largely missing for atypical species.

One of these atypical *Brucella* species is *B. inopinata*, which was unexpectedly isolated in 2008 from a 71-year-old woman in the United States with an endogenous breast implant infection and clinical signs consistent with brucellosis (De et al., 2008; Scholz et al., 2010). At this time, *B. inopinata* was the most genetically diverse *Brucella* species compared to the classical *Brucella* species. While all classical *Brucella* species are identical in their 16S rRNA and *recA* gene sequences, *B. inopinata* was the first to show multiple mutations in these genes (Scholz and Vergnaud, 2013; Scholz et al., 2016a). The comparative genomic analysis identified genomic regions that distinguished *B. inopinata* from the classic *Brucella* genomes, including one *B. inopinata* region comprising several genes coding for proteins associated with l-rhamnose utilization that have been shown to form the O-antigen component of the LPS in some bacteria (Giraud and Naismith, 2000; Wattam et al., 2012). Analysis of *Brucella* isolates from frogs and BO2 found that many of the genes required to generate the LPS in the traditional *Brucella* species (Al Dahouk et al., 2017) are lacking, but some of these strains had four genes associated with L-rhamnose utilization. Specifically, three of the frog isolates (B13-0095, 10RB9215, and 10RB9213) and the BO2 strain lacked many of the original genes but had the L-rhamnose utilization genes. Since that original analysis, these genes have been found in two additional genomes: *Brucella* sp. 141,012,304 (Eisenberg et al., 2017), which was isolated from a bluespotted ribbontail ray, and strain BO3 (Rouzic et al., 2021), a close relative of B13-0095 isolate, which was isolated from a human (Tiller et al., 2010).

In further studies addressing *B. inopinata* virulence, it was shown that *B. inopinata* is able to replicate intracellularly in macrophages and to cause disease and long-term infection in mice (Jimenez de Bagues et al., 2014; Al Dahouk et al., 2017). In contrast to classical *Brucella* species, *B. inopinata* BO1^T^ also caused death in the mouse model, which was not observed with classical *Brucella* species (Jimenez de Bagues et al., 2014).

Until now, *B. inopinata* BO1^T^ was the only existing isolate of this species. Here, we report the molecular characterization of a second *B. inopinata* strain isolated from a White’s Tree Frog (*Litoria caerulea*) in Switzerland. Comparative genome analysis clearly showed that strain FO700622 is a true member of *B. inopinata* and does not represent a *B. inopinata*-like organism, as previously reported for other atypical *Brucella* strains by other authors (Fischer et al., 2012; Scholz et al., 2016a). Within the last few years, exotic frogs have been recognized as an important host for atypical *Brucella* species worldwide (Shilton et al., 2008; Eisenberg et al., 2012; Fischer et al., 2012; Whatmore et al., 2015; Soler-Lloréns et al., 2016; Scholz et al., 2016a; Al Dahouk et al., 2017; Muhldorfer et al., 2017). Since exotic frogs are found in many zoos and are kept by exotic animal enthusiasts as terrarium pets and also provide a human food source, we discuss the possible public health implications of this finding.

## Materials and methods

### Case description

A female, captive White’s tree frog (*Litoria caerulea, synonym: Ranoidea caerulea,* natural habitat Australia and Papua New Guinea), with clinical signs of anorexia, fatigue, and a skin mass on its back suspicious of a skin abscess or neoplasia, was presented to a veterinary practice specialized in exotic animals. For 2 weeks, the frog showed progressive loss of appetite and inclined position in the water until it finally avoided swimming. After a general examination, swabs were taken from skin lesions for bacteriologic examination. As a precaution, the frog was given antibiotic treatment with Marbocyl FD (marbofloxacin 10 mg/kg, subcutaneously, daily for 10 days). The frog became increasingly lethargic and was euthanized 1 month after the first presentation and sent for autopsy.

The animal originated from a private breeder in Switzerland and was bought together with a second one of the same species in a pet shop in 2008. Both frogs were exclusively kept in a naturally decorated terrarium for nearly 10 years, from which they were rarely removed. Skin changes were noted on one frog 10 years after the initial purchase. The second frog remained clinically unremarkable and was moved to a second terrarium because of the diagnosis of a *Brucella* infection in the other frog. Both frogs were fed crickets and grasshoppers, and commercial calcium and vitamin powder was added regularly.

### Bacterial cultivation and preliminary identification

Following necropsy, bacteria were cultivated from various clinical samples (liver, spleen, lung, heart, kidney, ovary, gut, skin, and, in one case, intraocular fluid) on Columbia sheep blood (COLS COL+^2SBplus^; Oxoid, Wesel, Germany) and Endo-Agar (Becton Dickinson, Karlsruhe, Germany) at 37°C aerobically and with 5% CO_2_ for up to 96 h. For bacterial growth, the tissue samples were immersed in 70% ethanol, air-dried for 5 min, and sectioned with a scalpel. The cut surface was streaked over the agar plate. Skin swabs and fluids were applied directly to the solid culture media. Initial bacterial identification was done by MALDI-TOF analysis (Microflex LT, Bruker Daltonik GmbH, Bremen, Germany).

Subsequent phenotypic characterization of an isolate isolated from the skin (FO700622), sent to the *Brucella* Reference Laboratory of the Bundeswehr Institute of Microbiology in Munich, Germany, included growth on *Brucella* selective agar, Gram stain morphology, catalase and cytochrome oxidase activity, hydrogen sulfide (H_2_S) production, and biochemical characterization using API 20NE and API ZYM (bioMerieux, Nürtingen, Germany). *Brucella*-specific serological reactions with monospecific agglutination A and M antisera (Anses, Maisons-Alfort, France) were performed as described by Alton et al. (1988).

### Histo-pathology procedures

Histological examinations were performed according to a standard protocol. Samples were embedded in paraffin wax and stained with hematoxylin and eosin (HE). Periodic acidic Schiff (PAS) staining was performed for fungi and endoparasites, and a Ziehl–Neelsen stain was performed to detect acid-fast bacteria.

### Molecular identification by PCR

The detection of the *Brucella*-specific genetic markers *bcsp31* and *IS711* by real-time PCR and species-differentiating multiplex PCR (Bruce-ladder) was performed as described previously (García-Yoldi et al., 2006; Scholz et al., 2008b; López-Goñi et al., 2011). The 16S rRNA and *recA* gene sequences were determined and analyzed as described previously (Scholz et al., 2008a).

### Genome sequencing and assembly

High-quality genomic DNA (gDNA) was prepared for whole-genome sequencing by using the Qiagen genomic extraction kit and Qiagen Genomic-tip 20/G (Qiagen, Hilden, Germany) according to the manufacturer’s recommendations. DNA concentration was determined by the use of a Qubit® 2.0 Fluorometer (Thermo Fisher Scientific) and the Qubit® dsDNA high-sensitivity assay kit (Thermo Fisher Scientific).

Next, era® XT DNA Library Preparation kit (Illumina) with an input DNA amount of 1 to 3 ng was used for library preparation. Whole-genome sequencing was performed on a MiSeq instrument (Illumina) with corresponding MiSeq Reagent Kit v3 (600 cycles; 300 bp paired end) chemistry. A total of 20,866,712 sequencing reads were generated. Trimming of raw sequencing reads and *de novo* sequence assembly was performed using the software package CLC genomics workbench together with the microbial finishing module (Qiagen, Hilden, Germany). After quality trimming (quality limit 0.05, max ambiguities 2) and adapter removal, 20,865,498 reads remained with an average read length of 288.24 nucleotides, corresponding to an average coverage of approximately 1,900 × relative to the reference genome *B. melitensis* 16M^T^. Sequencing reads were down-sampled (reproducible sampling) to 5 M reads (approximately 200x coverage) and assembled *de novo* with a minimum contig length set to 500 bp. *De novo* assembly generated 40 assembled contigs (GC content 57.13%) with maximum and average lengths of 337.887 and 70.713 bp, respectively, and a genome length of 3,333,147 bp.

RAW sequencing reads were submitted to NCBI Sequence Read Archive, BioProject accession number: PRJNA906025. The assembled genome with additional information is publicly available in BV-BRC.^1^

### Scaffolding

Each of the 40 contigs produced when assembling *Brucella inopinata* strain FO700662 (Acc. No. JARQXC000000000; BioSample SAMN31890425) was blasted against the *B. suis* 1330 (Acc. No. AE014291, AE014292) genome to determine the order based on this reference. Contigs were manually combined into an artificial scaffold that matched the order in that genome.

### Calculating overall genome similarities

The average nucleotide identities between strain FO700622 (Acc. No. JARQXC000000000; BioSample SAMN31890425) and *B. inopinata* BO1^T^ (Acc. No. ADEZ00000000) as well as its closest relatives, strains BO2 (Acc. No. CP065399, CP065400) and BO3 (Acc. No. CP047232, CP047233), were determined using the online tool (OrthoANIu method; Yoon et al., 2017) form EzBioCloud available from https://www.ezbiocloud.net/tools/ani.

Genome similarities (DDH estimates) between *B. inopinata* BO1^T^ and strains BO2 and BO3 were also calculated using the genome-to-genome distance calculator^2^ (Meier-Kolthoff et al., 2022).

### *In silico* MLVA and MLSA

*In silico* MLVA-16 analysis (Le Flèche et al., 2006; Al Dahouk et al., 2007) was carried out as described previously using an in-house script (Georgi et al., 2017). Each locus was checked in respect of the expected total length, internal repeat homogeneity, or probability to get collapsed VNTRs during the assembly. The resulting MLVA-16 genotype of strain FO700622 was compared to entries of a public database consisting of more than 6,000 *Brucella* strains of each species that can be assessed online via http://microbesgenotyping.i2bc.paris-saclay.fr/. Extended multilocus sequence analysis (BruMLSA21) based on 21 different markers was carried out as described by Whatmore et al. (2016). The scheme and the database are available at PubMLST.^3^ For *in silico* analysis, the scheme was downloaded from the database and a task template was created with SeqSphere+ software, v5.0.90 (Ridom GmbH, Münster, Germany). The required identity for the target scan was set to 90% with the required 99% alignment to the respective reference gene. Automatic allele calling from assembled genomes was done using SeqSphere+.

### Phylogeny and comparative analyses

Thirty-three *Brucella* genomes (Supplementary Table 1) were used in a comparison. All genomes, including the new isolate FO700662, were annotated in PATRIC, now known as BV-BRC, the Bacterial and Viral Bioinformatics Resource Center (Wattam et al., 2017, 2018; Davis et al., 2020; Olson et al., 2022) using the RASTtk pipeline (Brettin et al., 2015) which includes annotated genes being automatically assigned into two kmer-based protein families that are genus-specific (PLFams), or are global spanning the genus boundary (PGFams; Davis et al., 2016). The genome of FO700662 is available in BV-BRC under the identifier 1218315.22. Five *Ochrobactrum* reference genomes were also included in the phylogeny and were used to verify *Brucella*-specific genes (Supplementary Table 2).

The phylogenetic trees were generated using the Codon Trees pipeline at BV-BRC. Single-copy PGFams present in each genome were identified, and the protein (amino acid) and gene (nucleotide) sequences were used for each of the selected genes. The tree was generated using MUSCLE (Edgar, 2004; amino acid alignment), Biopython (Cock et al., 2009; codon alignment), and RaxML (Stamatakis et al., 2008; Stamatakis, 2014) for tree generation using the GTRGAMMA model for nucleotides and the LG model for amino acids. Support values were generated using 100 rounds of the “Rapid” bootstrapping option (Stamatakis et al., 2008) of RaxML. The resulting Newick file was visualized using FigTree (Rambaut, 2006).

Both the 40 contigs and the reference-based scaffolded FO70062 genome were compared to the *B. suis* 1330 genome using the Proteome Comparison tool to identify areas within the genomes that were not shared with the comparison genome(s). In addition, protein families were arranged based on the order that they occurred in specific genomes (*B. suis* 1330, *Brucella* sp. 09RB8471, and *Brucella* sp. FO700662) to look for unique regions or regions of potential lateral transfer in the amphibian isolates using the Protein Family Sorter tool (Wattam et al., 2018). Once a potential genomic island was identified, the region of the genome was examined in JBrowse (Skinner et al., 2009), with the flanking regions examined for tRNA genes. Flanking regions were confirmed using the Proteome Comparison tool (Overbeek et al., 2014). Each potential island was visualized in BV-BRC’s Compare Region Viewer (Overbeek et al., 2014) to look for conservation of the gene neighborhood across genomes that had a similar region, and the presence or absence in specific genomes was confirmed by BLASTN of the regional sequence against the target genomes with an expected value of 0.0001, sc_match of 1, sc_mismatch of −2, gap_open of 0, gap_extend of 0, and filter of L;m; (Boratyn et al., 2013). Each region was also searched against a database of plasmid genomes in BV-BRC (Wattam et al., 2017) and also against the reference and representative genomes in the genus *Ochrobactrum* to confirm whether novel regions were conserved from a recent ancestor. Genes in these regions that were not annotated as hypothetical were examined to see whether they were present in KEGG pathways (Kanehisa et al., 2023) and subsystems (Overbeek et al., 2005) or were described in the published literature.

### Core-genome-based MLST

A previously developed core-genome-based MLST (cgMLST) assay (Janowicz et al., 2018) using SeqSphere+ software, v5.0.90 (Ridom GmbH, Münster, Germany) was used to determine the genetic relationship of strain FO700622 and BO1^T^. The cgMLST scheme covers 2,704 genes with a total of 2.441.649 out of 3.294.931 bp (74%) of the reference strain *B. melitensis* 16M^T^ (NC_003317.1; NC_003318.1) and can be freely downloaded from the cgMLST Nomenclature Server^4^ or within the software. The assay uses a required identity for the target scan of 90% with a required 100% alignment to the respective reference genes. For cgMLST analysis, the *de novo* assembly generated by the CLC Genomic Workbench was imported as a FASTA file into SeqSphere+. The type strains of all currently known *Brucella* species as well as a set of biovar reference strains were included in the analysis. Genome accession numbers are given in Supplementary Table 1.

## Results

### Bacterial cultivation and identification

After 48 h of culture, an almost uniformly mixed bacterial flora of different non-fermenters was observed. In addition, *Brucella*-suspicious colonies were isolated in high grade from the skin but also in small numbers from all clinical specimens tested. Bacteria were positive for cytochrome oxidase and catalase with a rapid urease reaction (5 min). No hemolysis was observed. Bacterial identification using MALDI-TOF revealed *Ochrobactrum* sp. with a high identification score of 2.2. However, with the knowledge of the recent emergence of atypical *Brucella* in exotic frogs resembling *Ochrobactrum*, one sample (FO700622) was sent to the Institute of Microbiology, Munich, for further clarification. Strain FO700622 was highly similar to *B. inopinata* BO1^T^ (De et al., 2008; Scholz et al., 2016a) in all phenotypic reactions. Similar to *B. inopinata* BO1^T^, rapid growth was observed on all media tested in a temperature range of 28°C–40°C. On *Brucella* agar (Merck, Darmstadt), growth became visible within 10 h of incubation at 37°C with or without supplementary CO_2_. Single colonies of 1–2 mm were formed within 24 –48 h. Weak agglutination was observed with monospecific anti-M serum up to a dilution of 1:40 but not with anti-A serum. Production of H_2_S and Voges–Proskauer reaction is positive. Strains were negative for hydrolysis of esculin, gelatin liquefaction, production of indole, and citrate utilization. Strains tested positive (API ZYM) for acid phosphatase, alkaline phosphatase, trypsin, leucine arylamidase, and naphthol-AS-BI-phosphohydrolase. Strains were negative (API ZYM) for esterase, esterase lipase, lipase, valine arylamidase, cystine arylamidase, α-chymotrypsin, α- and β-galactosidase, β-glucuronidase, α- and β-glucosidase, N-acetyl-β-glucosaminidase, α-mannosidase, and α-fucosidase. Strains tested positive (API 20NE) for d-glucose, maltose, l-arabinose, d-mannose, N-acetylglucosamine, and adipic acid and negative for d-mannitol, citric acid, gluconate, capric acid, malic acid, and phenylacetic acid. In API 20E, Strains tested positive for fermentation of l-arabinose.

### Pathological findings

**Figure 1:**
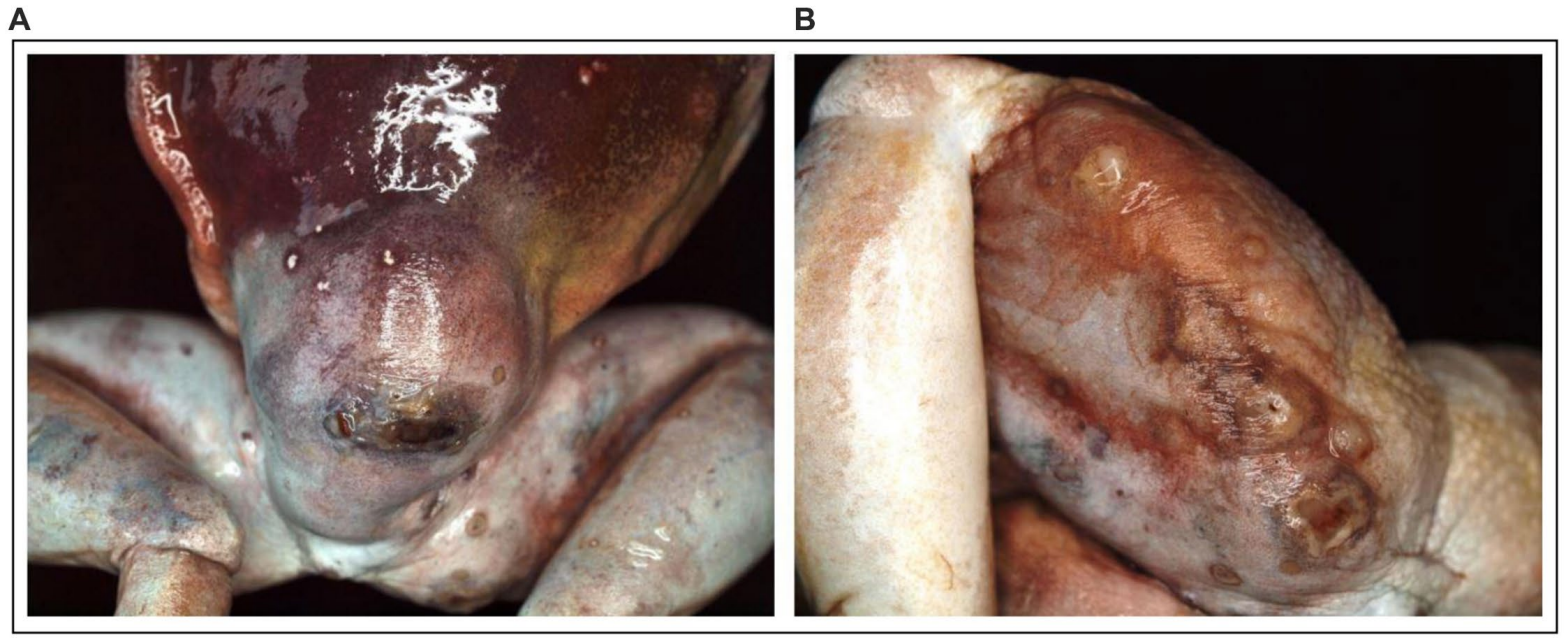
**(A)** Dermal granulomatous inflammation with excoriations of the skin measuring 3.0 × 3.0 × 1.3 cm from a 13-year-old White’s tree frog after fixation. **(B)** Diffuse swelling of the thigh with an excoriation on a 13-year-old White’s tree frog after fixation.

The entire body of the dead White’s tree frog (*Litoria caerulea*), with a body size of 11.8 cm and a body weight of 112 g, was submitted for pathological examination. Gross examination showed a dorsal mass of 3.0 × 3.0 × 1.3 cm cranial to the cloaca (Figure 1A). A 0.6 cm large excoriation was observed on the right front limb. Both thighs were diffusely moderately swollen, and there was a 0.3 cm large excoriation on the right thigh (Figure 1B). The internal organs were macroscopically unremarkable as far as could be evaluated due to their fixation in formalin.

Histopathologically, the skin lesions on the thighs and the mass on the back showed moderate epidermal hyperplasia with multiple ulcerations (Supplementary Figure 1).

**Figure 2:**
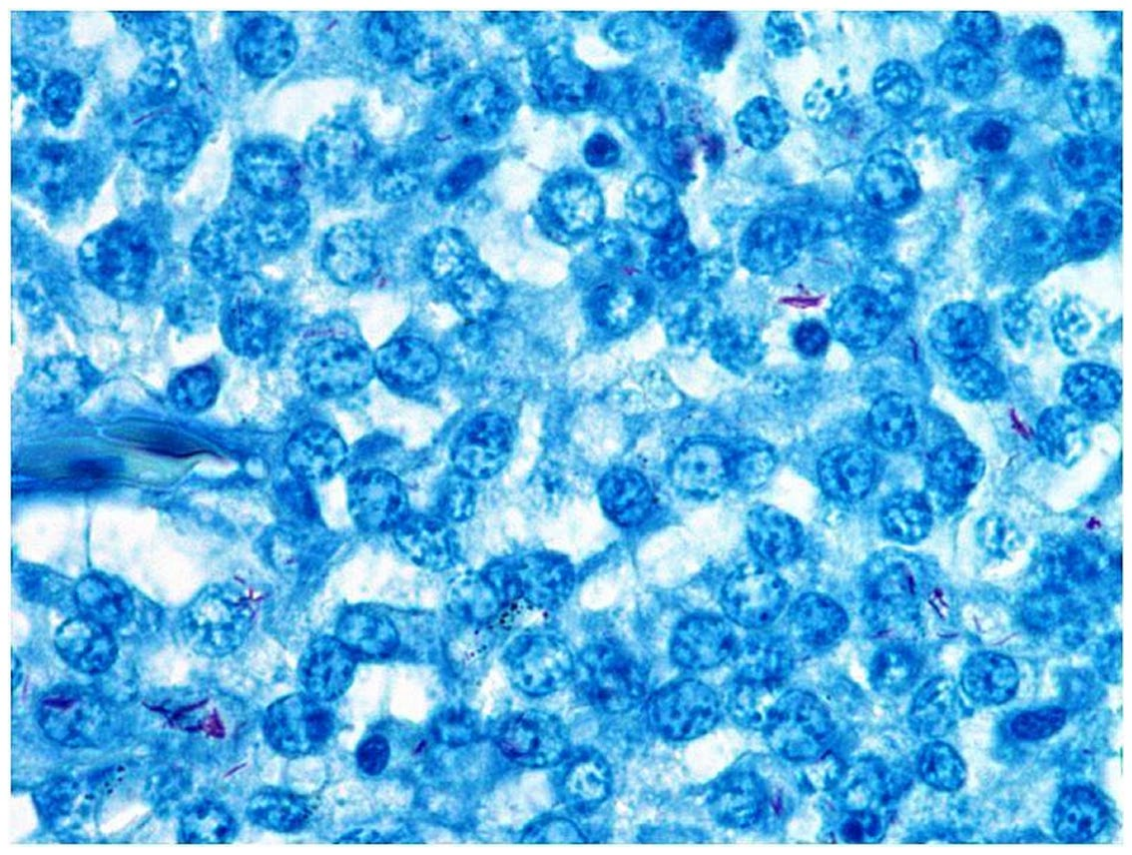
Granulomatous inflammation composed of macrophages and single lymphocytes. Moderate numbers of intralesional acid-fast, rod-shaped bacteria phagocytosed by macrophages or freely located (Ziehl–Neelsen, 1,000x magnification).

A severe multifocal to coalescing granulomatous inflammation with moderate numbers of intralesional acid-fast, rod-shaped bacteria in the macrophages and freely located were seen in the dermis and the underlying musculature (Figure 2).

The musculature contained moderate numbers of cysts of cestodes. The morphology of the parasite was indicative of the sparganum of *Spirometra erinacei* (Supplementary Figure 2). Other organs examined were morphologically unremarkable.

### PCR and *in silico* analysis

Strain FO700622, received as *Ochrobactrum* sp. as identified by MALDI-TOF analysis, was positive in PCR for the *Brucella*-specific targets *IS711* and *bcsp*31, suggesting its affiliation to the genus *Brucella* but not to *Ochrobactrum*. The species-differentiating Bruce-ladder-multiplex-PCR revealed the *B. inopinata* BO1^T^-specific banding pattern with fragment sizes of 152, 272, 450, 587, 794, and 1,682 base pairs, indicating its affiliation or close relationship to this species. This finding was remarkable because up to that date, all *Brucella* isolates from exotic frogs had a unique banding pattern, different from all other species, consisting of five fragments with sizes of 152, 272, 450, 587, and 794 bp (Eisenberg et al., 2012). We, therefore, genome-sequenced strain FO700622 for further molecular characterization. The *in silico* generated MLST-21 profile of strain FO700622 was identical to the specific profile of *B. inopinata* BO1^T^ (sequence type 69), confirming that strain FO700622 belongs to this species. In contrast, the profile of the to date closest related strain BO2 differed in all of the given markers (*mviM-*negative, no assigned ST).

With the exception of one VNTR marker (bruce 18), the MLVA profiles of *B. inopinata* BO1^T^ and strain FO700622 were identical, whereas the closest relative, BO2, differed in six of the sixteen markers. MLVA-16 profiles were as follows: *B. inopinata* BO1^T^ (2, 5, 9, 13, 3, 2, 5, 4, 8, 40,0, 10, 0 3, 3, and 0); *B. inopinata* FO700662 (2, 5, 9, 13, 3, 2, 5, 4, 9, 40, 0, 10, 0, 3, 3, and 0), and *Brucella* sp. BO2 (1, 5, 3, 13, 4, 2, 5, 3, 12, 37, 8, 19, 5, 3, 5, and 10). When calculating the genome-to-genome distance, the DNA–DNA hybridization (DDH) estimate between strains BO1^T^ and FO700662 was 99.4%, indicating that these genomes are highly similar. In comparison, the DDH estimate between strain BO1^T^ and BO2 was 85.8% and 84.9% with strain BO3. This was also reflected in the ANI values obtained (BO1^T^/FO700662: 99.86; BO1^T^/BO2: 98.31; BO1^T^/BO3: 98.25) with genome coverages of 75.14%/74.35, 70.76%/68.97, and 64.89%/64.11%, respectively.

### Phylogeny

**Figure 3:**
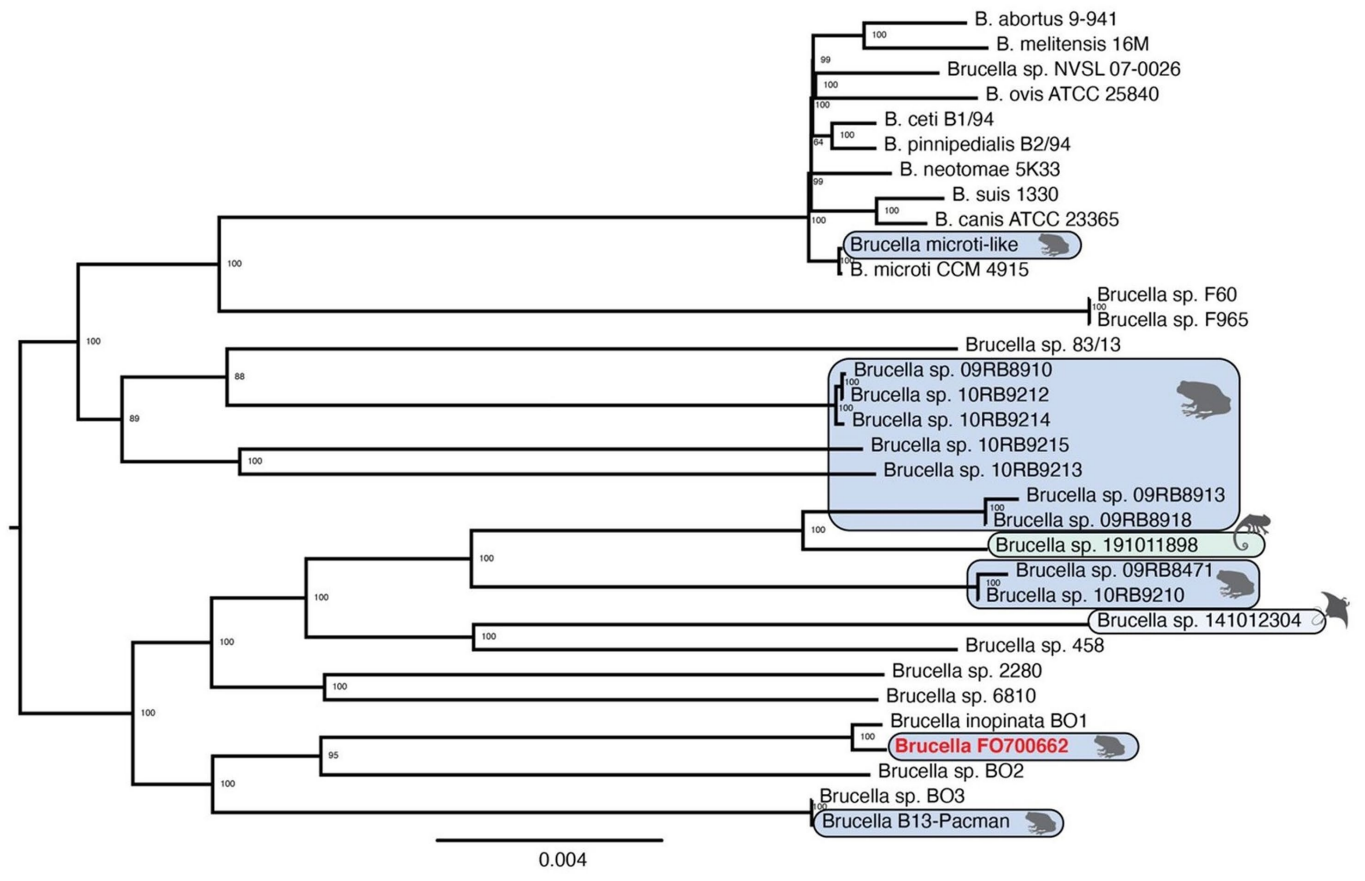
Midpoint rooted, maximum likelihood tree for 33 *Brucella* genomes.

The phylogenetic tree was built using the Codon Trees pipeline at PATRIC. Both the amino acid and nucleic acid sequences from 901 single-copy orthologous genes included 260,286 amino acids and 780,858 nucleotides that were concatenated together in an alignment and used to generate the tree (Figure 3).

The *B. inopinata* BO1^T^ strain (De et al., 2008), an isolate from human infection, was closest to the FO700622 strain, with a 100% support value. The closest strain to these two genomes was another human isolate, the BO2 strain (Tiller et al., 2010). The branch containing FO700622 and the two human isolates is found on the same branch as *Brucella* strains that have been recently isolated from the Pacman frog (strain B13-0095) and BO3, a human isolate (Rouzic et al., 2021). The next closest clade includes an isolate from the bluespotted ribbon tail ray (strain 141,012,304; Eisenberg et al., 2017), other African bullfrog isolates (strains 10RB9210, 09RB8471, 09RB8918, and 09RB8913; Al Dahouk et al., 2017), an isolate from a chameleon (strain 191,011,898; Eisenberg et al., 2020), and three isolates from humans in Australia (strains 6,810, 2,280, and 458). Genetic analysis does not suggest a clear distinction between *Brucella* strains isolated from warm- or cold-blooded hosts as isolates from cold-blooded hosts appear in both ancestral and classical clades of the phylogenetic tree. The *Brucella microti*-like genome was isolated from a *Pelophylax ridibundus* in a domestic frog farm in France (Jay et al., 2018) and was found within the classical clade (Figure 3). The addition of *Ochrobactrum* genomes (Supplementary Figure 3) shows that the root of the *Brucella* tree occurs in the middle of the atypical genomes and is not clear between the classical and atypical strains.

### Core-genome-based MLST

The cgMLST assay was originally optimized for *B. melitensis*; however, the high genetic similarity of all *Brucella* species, including atypical *Brucella*, allows accurate typing of atypical *Brucella*. Only 10% to 15% of the 2,704 target genes cannot be used, thus, there is still an average of 2,300 genes available for cgMLST analysis.

**Figure 4:**
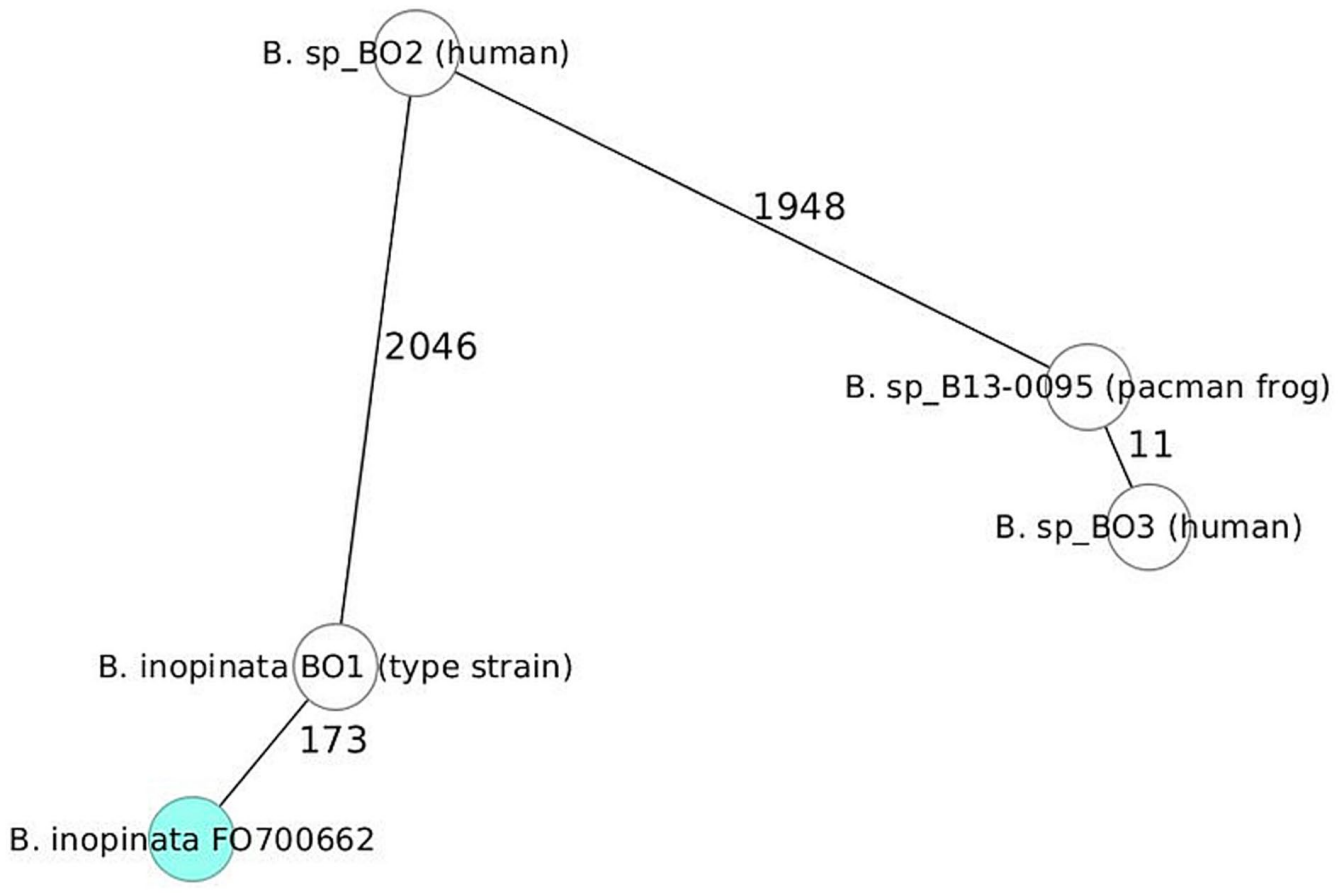
cgMLST minimum spanning tree (MST). Direct comparison of strains BO1^T^, BO2, FO700622, BO3, and B13-0095. Allele distances are shown in numbers.

The minimum spanning tree (MST) was generated with the SeqShere+ software. The genomes of genetically atypical *Brucella* species are well separated from the classic *Brucella* species by 1,969 allelic differences with *B. ovis* lying on the path between the two groups (Supplementary Figure 4). The distances among most genetically classical species ranged from 730 (*B. pinnipedialis* / *B. ceti*) to 1,719 alleles (*B. pinnipedialis* / *B. melitensis*). The close genetic relationship between *B. canis* and *B. suis* bv 4 was reflected by a distance of only 308 alleles (lower left). Significantly larger distances (>2,000 alleles) were detected in epidemiologically unrelated isolates of the atypical group, indicating higher genetic diversity in this population compared to the classical *Brucella* species. Only epidemiologically related frog isolates were clustered together with few allelic differences. The distance of 36 alleles between two strains of Australian rodents (NF2651 and 83/13) supports a possible epidemiological link. The two *B. vulpis* strains (F60H and F965) isolated from two different red foxes in Lower Austria differed in only 15 alleles. Only 173 different alleles were detected between *B. inopinata* BO1^T^ and *B. inopinata* strain FO700662, confirming their very close genetic relationship. Interestingly, strain BO2, which until then was considered the closest relative of strain BO1^T^, differed in 2,046 alleles in a head-to-head comparison (Figure 4). A similarly large distance of 1,948 alleles was observed between strains BO2 and B130095/BO3. It is noteworthy that strain BO3, a human isolate, differed in only 11 alleles in cgMLST analysis from strain B13-0095 isolated from a Pacman frog. An allelic distance matrix of strains FO700662, BO1^T^, BO2, BO3, and B130095/BO3 is given in Supplementary Table 3.

### Distinctive regions

Seventeen regions of interest were identified when all strains were compared to FO700662 (Table 1). A complete list of the genes in these regions is available (Supplementary Table 4). A BLAST analysis of the genomic sequences of each of these regions showed that they were not present in the genomes associated with the “classical *Brucella*” strains and also were not universally shared across all of the strains found in the non-classical *Brucella* strains that are found in the lower half of the phylogenetic tree (Table 2; Figure 3).

**Table 1:** Genomic regions unique to FO700662 when compared to classical *Brucella* strains.

Region	Contig	Start	Stop	Size	tRNA	Genes
One	Contig 20	193,697	199,385	5,688	Yes	10
Two	Contig 39	247	18,081	17,834	No	19
Three	Contig 16	18,004	21,422	3,418	No	7
Four	Contig 12	109,977	113,564	3,587	Yes	6
Five	Contig 2	196,970	200,851	3,881	No	6
Six	Contig 7	211,494	224,602	13,108	Yes	15
Seven	Contig 2	52	12,378	12,326	Yes	16
	Contig 37	41,518	53,206	11,688	Yes	11
Eight	Contig 15	65,238	72,094	6,856	No	11
Nine	Contig 28	253	26,954	10,795	No	9
Ten	Contig 36	601,007	628,634	26,701	No	26
Eleven	Contig 36	89,967	91,628	1,661	Yes	5
Twelve	Contig 22	123,522	127,301	3,779	Yes	8
Thirteen	Contig 9	205,073	236,210	31,137	No	20
Fourteen	Contig 9	236,212	270,742	34,530	Yes	52
Fifteen	Contig 4	52	16,348	16,296	Yes	8
Sixteen	Contig 4	32,067	63,489	31,422	No	31
Seventeen	Contig 26	36,035	45,309	9,274	No	10

**Table 2:** Genomic regions unique to FO700662 with more than 50% query coverage in the non-classical *Brucella* strains.

Strain	Host	1	2	3	4	5	6	7	8	9	10	11	12	13	14	15	16	17
09RB8910	Amphibian													78.1				
10RB9212	Amphibian				56.7									78.1				
10RB9214	Amphibian													78.1				
10RB9215	Amphibian									100				81.9				100
10RB9213	Amphibian									100				81.8				100
09RB8913	Amphibian	85										100		52.8				
09RB8918	Amphibian	85										100		52.8				
191,011,898	Reptile	85										100		78.1				100
09RB8471	Amphibian	86.5			90.1									81.8				100
10RB9210	Amphibian	86.5			60.7									81.8				100
141,012,304	Ray	69.8								100		90.3		52.8				100
458	Human		100						100	100				88.6		50.4		100
2,280	Human	85.6	100		51.3				100	51		77.2		88.6		50.4	100	
6,810	Human	97.7		70.1	60.8				100			77.2						
BO1	Human	100	100	100		66.2	96.9		100	100	65.7	100	100	86.3		68	50.4	100
FO700662	Amphibian	100	100	100	100	100	100	100	100	100	100	100	100	100	100	100	100	100
BO2	Human	100	100		59.1				100	100				88.6		90.2	90.7	100
BO3	Human	100								100		77.2		78.2				
B13-Pacman	Amphibian	100								100		77.2		78.2				

The combined regions include nine tRNA genes and 264 protein-coding sequences, 139 (52.6%) of which are annotated as hypothetical. Seven of the regions (Regions 1, 3, 4, 6, 10, 12, and 14) have at least one phage gene annotated within it. Two of the regions (Regions 7 and 14) were unique to FO700662, but the others are either narrowly (Regions 4, 5, 6, 10, and 12) or widely (Regions 1, 13, and 17) shared across the non-classical strains. Many of these regions (1, 4, 6, 7, 11, 12, 14, and 15) are flanked by tRNA genes, which are known to be associated with horizontal gene transfer (Hacker and Kaper, 2000; Ochman et al., 2000). Other indications of horizontal transfer included the presence of genes annotated as mobile element proteins or transposases, and several of the regions have one or more genes that are identified as such (Regions 1, 2, 4, 6, 7, 10, 12, 13, and 16). Each region was compared to the plasmid database using BLASTN in BV-BRC (Davis et al., 2020). Five regions (Region 8, 9, 11, 16, and 17) had more than a 10% query coverage match against plasmid genomes in that database (Supplementary Tables 4, 5), indicating that they could have been incorporated by lateral transfer. These regions were all on the second chromosome.

BLASTN analysis of the nucleotide sequences of the regions was compared to the five *Ochrobactrum* representative genomes. Region 5 had a query coverage of 87% query coverage to *O. rhizosphaerae* SJY1, and region 14 had 67% to *O. intermedium* LMG 3301, indicating that these two regions were ancestral and did not enter *Brucella* by lateral, or horizontal transfer. Region 11 had 38% query coverage when compared to *O. anthropi* ATCC 49188, but this was one of the plasmids in this genome.

While most of the 272 genes found in these 17 regions are annotated as hypothetical (Supplementary Table 4), several regions contain some notable genes.

Regions 1, 3, 4, 6, 10, 12, and 14 all have bacteriophage genes annotated, with some regions (3, 6, and 14) having a number of these genes present.

**Figure 5:**
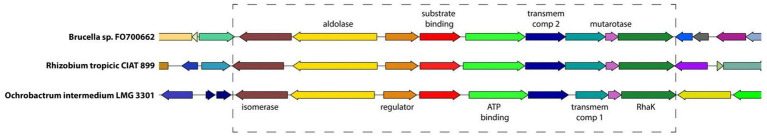
Genes from region nine, depicted in the box, showing conservation of gene length and orientation in the FO700662 compared to the *Rhizobium* plasmid and *Ochrobactrum* contig in which they were also found.

All the genes in Region 9 are involved in rhamnose metabolism, with some being potentially active in the KEGG Fructose and Mannose pathway. Four of these genes are predicted to work as a rhamnose transporter (Richardson and Oresnik, 2007). This region had weak homology when compared to *O. intermedium* LMG 3301 (43%) and also in a plasmid found in *Rhizobium topicic* (39%). An examination of the genes found in this region showed that the genes and their orientation were strongly conserved in these particular genomes (Figure 5), with differentiation in the flanking regions. This was the most strongly conserved of the regions with non-*Brucella* genomes.

Region 11 has a toxin/antitoxin HigB/HigA system. These types of systems have been found in many pathogens (Wood and Wood, 2016). Region 16 has three genes (proVWX) that have been identified as the *proU* operon in other bacteria, which encodes a binding protein-dependent transport system that is essential for the uptake of osmoprotectants such as glycine betaine and is known to be upregulated in response to osmotic stress (Lucht and Bremer, 1994).

A close examination of the genes that are known or predicted to be involved in lipopolysaccharide (LPS) production was conducted. This included the *wbk* region, *wboA* and *wboB*, and the four genes in BO2 (Wattam et al., 2012) that other bacteria use for making a rhamnose-based O-antigen (Table 3).

**Table 3:** BLAST results showing genes with more than 50% identity to LPS amino acid sequences.

Strain	Host	Rhamnose genes				Wbo genes		Wbk genes												
		rfbD	rfbB	rfbC	rfbA	wboB	wboA	wbkE	manA	manC	manB	wbkA	gmd	per	wzm	wzt	wbkB	wbkC	wbkF	wbkD
09RB8910	Amphibian																		97	100
10RB9212	Amphibian																		97	100
10RB9214	Amphibian																		97	100
10RB9215	Amphibian	94	98	98	98														98	99
10RB9213	Amphibian	94	98	98	97														97	100
09RB8913	Amphibian																		96	100
09RB8918	Amphibian																		96	100
191,011,898	Reptile																		96	100
09RB8471	Human																		98	99
10RB9210	Human																		98	99
141,012,304	Ray	96	98	98	99														98	100
458	Human	95	99	99	98														98	99
2,280	Human							99	98	99	92								97	99
6,810	Human							99	99	99	93	pseudo							100	100
BO1	Human					100	100	100	100	100	100	100	100	100	100	100	100	99	100	100
FO700662	Amphibian					100	100	100	100	100	100	100	100	100	100	100	100	100	100	100
BO2	Human	100	100	100	100														97	99
BO3	Human	97	98	98	98														98	99
B13-Pacman	Amphibian	97	98	98	99														98	99

Both BO1^T^ and FO70062 share the 13 genes in the *wbk* region that are essential for lipopolysaccharide (LPS) synthesis (Godfroid et al., 2000; Gonzalez et al., 2008; Al Dahouk et al., 2017), as well as *wboAB*. Both strains are missing the rhamnose genes first identified in BO2. An expanded analysis, using the protein sequences for the BO2 rhamnose genes and WboAB and the Wbk proteins from FO700662, showed that other than BO2, only six of the 19 non-classical strains have the four rhamnose genes (10RB9215, 10RB9213, 1412304, and BO3 and B13 Pacman). WbkF and WbkD are shared across all strains. The other genes in the Wbk region are absent from most strains, except for WbkE, ManA, ManC, and ManB, which are strains 2280 and 6810. Strain 6810 is also a pseudogene that matches WbkA.

## Discussion

While our understanding of the *Brucella* genus remained unchanged for decades in the past, several new *Brucella* species and strains of human origin and from various new animal hosts have been described more recently (Godfroid et al., 2005; Foster et al., 2007; Scholz et al., 2008b; Whatmore et al., 2014; Scholz et al., 2016b; Eisenberg et al., 2017). The isolation from cold-blooded hosts, particularly amphibians but also from fish and reptiles, has greatly expanded the host range of this medically important genus. Exotic frogs, in particular, have become an important newly recognized host of *Brucella* in recent years (Shilton et al., 2008; Eisenberg et al., 2012; Fischer et al., 2012; Soler-Lloréns et al., 2016; Muhldorfer et al., 2017). Isolation from different continents indicates a worldwide distribution of atypical *Brucella* in different exotic frog species (Scholz et al., 2016a). The majority of infections induce severe clinical signs in the frogs and frequently lead to death (Muhldorfer et al., 2017). However, it is currently unknown whether the mucous skin of frogs forms a natural reservoir for *Brucella* and whether the disease only occurs after trauma or stress conditions. The infection could also occur through contaminated food or an unknown reservoir in soil or water. Isolates from exotic frogs belong to the so-called atypical *Brucella* which are phenotypically close to *Ochrobactrum* spp., a soil-associated facultative human pathogen, but genetically more closely related to the *Brucella* genus (Lebuhn et al., 2000; Elsaghir and James, 2003; Scholz et al., 2008a). In-depth molecular-biological analyses at the isolate level have shown that brucellae from exotic frogs, in contrast to classic brucellae, have a markedly higher level of genetic diversity and possess genes on their chromosomes from other soil-associated bacteria, indicating horizontal gene transfer (Wattam et al., 2009; Occhialini et al., 2022).

Although most atypical brucellae are currently being isolated from amphibian hosts, the first genetically (and phenotypically) atypical *Brucella* strain BO1, later named *B. inopinata*, was not isolated from a frog but unexpectedly from a 71-year-old woman in 2008 with an endogenous breast implant infection (De et al., 2008). At this point, however, the source of infection was unknown and exotic frogs had not yet been recognized as hosts for atypical *Brucella*. The first isolation of atypical brucellae from wild-caught African bullfrogs (*Pyxicephalus edulis*) from Tanzania was published in 2012 (Eisenberg et al., 2012). Eight-locus MLSA (Whatmore et al., 2007) placed the isolates close to *B. inopinata* BO1^T^ and other atypical isolates from Australian rodents (Eisenberg et al., 2012) forming a new branch distinct from the classic *Brucella* species but clearly related to *Brucella* and more distantly related to *Ochrobactrum*. To date, the number of atypical brucellae (sometimes referred to as “non-core” brucellae) has increased significantly due to the description of various new atypical isolates from different sources (Al Dahouk et al., 2017; Muhldorfer et al., 2017; Whatmore and Foster, 2021). Because *B. inopinata* was the first atypical species described (Scholz et al., 2010) and was still the only validly published species among the atypical brucellae, members of the atypical clade are often referred to as “*B. inopinata*-like.” However, as shown in the cgMLST analysis targeting 2,704 genes (Figure 4; Supplementary Figure 4), even to date most closely related atypical strain BO2, also a human clinical isolate, differs in 2,046 alleles from *B. inopinata* BO1^T^, while the strain analyzed in this study (FO700622) differs from *B. inopinata* BO1^T^ in only 173 alleles. This close and unexpected proximity of *B. inopinata* BO1^T^ and strain FO700622 prompted us to perform a more detailed comparative genomic analysis of both strains and other atypical members and to investigate whether strain FO700622 is a true member of *B. inopinata*. Since strain BO2 was identified as the genetically most closely related strain compared to BO1^T^ in several previous studies (Wattam et al., 2012; Al Dahouk et al., 2017), the distinguishing features of both strains were investigated in detail.

Strain FO700622 showed the specific banding pattern of *B. inopinata* BO1^T^ in the species-differentiating Bruce-Leader PCR. Both MLSA and *in silico* MLVA also confirmed strain FO700622 as a true member of *B. inopinata* with an identical MLSA profile and only one repeat difference in one of the 16 VNTR markers. In contrast, strain BO2 differed significantly from strain BO1^T^ and differed in all MLST markers and six VNTR markers. The calculated genome-to-genome distance of 99.4% between strains BO1^T^ and FO700622 showed that both genomes are highly similar while comparing strain BO1^T^ with BO2 a significantly lower value of 85.8% was obtained.

The phylogenetic tree (Figure 3) clearly shows that FO700622 and *B. inopinata* are most closely related. Strain BO2 is close to both of these strains, but it is also distinctly different. Most notably, it is missing the *wboAB* and the genes in the Wbk region that are essential in producing the O-antigen and, in the same location, has four genes that are involved in the formation of the O-antigen component of the LPS in many gram-negative bacteria (Giraud and Naismith, 2000; Wattam et al., 2012).

While the tree does show that the previously known “classical” strains are united in a clade, the genomes isolated from cold-blooded hosts are not. The *Brucella microti*-like strain is comfortably located within the classic clade, and it was isolated from a frog. When *Ochrobactrum* genomes were included in the phylogeny (Supplementary Figure 3), the root of the *Brucella* genomes is found somewhere within the middle of the atypical genomes. This suggests that early radiation of the *Brucella* had several successful lines, with one particular line being very successful and evolving into the ancestral genome that is the progenitor of the classic clade.

The identification of strain FO700622 as a second *B. inopinata* isolate and its isolation from an exotic frog raises the question of whether amphibians may play a role as a potential source of human infection. This could apply to people who keep exotic frogs as pets in a terrarium, or to frogs that are produced for human consumption. Indeed, only recently, another member of the biochemically atypical *Brucella*, *B. microti*, was isolated in large quantities from frogs bred for human consumption at a French frog farm (Jay et al., 2020). While most atypical *Brucella* are not risk classified, *B. inopinata* has been classified in risk group 3. It needs to be clarified how the natural occurrence of a risk group 3 pathogen in exotic frogs kept as pets or housed in zoos is to be assessed.

However, it should be noted that human infections with atypical *Brucella* are extremely rare with only four cases reported to date (Tiller et al., 2010; Soler-Lloréns et al., 2016; Rouzic et al., 2021). In 2010, strain BO2 was isolated from a patient in Australia with severe pneumonia (Tiller et al., 2010), and more recently in 2019, strain BO3 almost identical to strain B13-0095 isolated from a Pacman frog in Texas, United States, was isolated from a French patient with typical signs of severe brucellosis. The patient infected with strain BO3 had close contact with exotic animals including Pacman frogs (Rouzic et al., 2021). In cgMLST analysis (Figure 4; Supplementary Figure 4), strains BO3 and B13-0095 were nearly indistinguishable with a difference of only 11 alleles, which is even closer than the relatedness of strains BO1^T^ and FO700662. This is remarkable in view of the overall higher genetic diversity among the atypical *Brucella* and no obvious epidemiological connection. Three other clinical isolates of *B.* sp. 2,280 (biosample: SAMN12091575), *B.* sp. 6,810 (Biosample: SAMN15962648), and *B.* sp. 458 (BioSample: SAMN18395631) were recently isolated from human patients in Australia, with no further information available. However, in Australia, atypical *Brucella* have been isolated from rodents and repeatedly from exotic frogs (Tiller et al., 2010; Latheef et al., 2020).

The worldwide occurrence and high genetic diversity of atypical *Brucella* isolated from various exotic frog species indicate that amphibians may play an important role as natural reservoirs and potential vectors of “atypical” *Brucella* species and also may function as a source for human infections. As pointed out previously (Scholz et al., 2016a), we speculate that atypical *Brucella* may have a reservoir in the soil rhizosphere or in yet unknown non-vertebrate hosts occasionally colonizing the skin of amphibians as opportunistic pathogens. Stressful conditions during transport, especially when importing wild-caught animals or after skin injuries that may occur during quarantine or improper animal husbandry, might trigger local and systemic infection.

## Data availability statement

The datasets presented in this study can be found in online repositories. The names of the repository/repositories and accession number(s) can be found in the article/Supplementary material.

## Ethics statement

Ethical review and approval was not required for the animal study because bacterial isolation from the frog was done as routine diagnostics in a veterinary laboratory. Written informed consent was obtained from the owners for the participation of their animals in this study.

## Author contributions

HS and AW conceived and designed the experiments related to molecular strain characterization, analyzed all molecular data, and wrote the original manuscript. SA contributed to conception and design of the study and wrote sections of the manuscript. KH and PS carried out all experiments regarding dissection of the frog, including histo-pathological examinations and initial isolation of bacteria and also wrote the accompanying text in the manuscript. DG-D was involved in the initial strain isolation and further characterization. All authors contributed to the article and approved the submitted version.

## Funding

AW was funded in part by Federal funds from the National Institute of Allergy and Infectious Diseases, National Institutes of Health, Department of Health and Human Services, under contract no. 75N93019C0007. The project on which this report is based was partially funded by the Robert Koch Institute with funds from the Federal Ministry of Health under funding number 1369-448.

## Conflict of interest

KH and DG-D were employed by LABOklin GmbH and Co KG, Bad Kissingen, Germany.

The remaining authors declare that the research was conducted in the absence of any commercial or financial relationships that could be construed as a potential conflict of interest.

## Publisher’s note

All claims expressed in this article are solely those of the authors and do not necessarily represent those of their affiliated organizations, or those of the publisher, the editors and the reviewers. Any product that may be evaluated in this article, or claim that may be made by its manufacturer, is not guaranteed or endorsed by the publisher.

## Footnotes

^1^ https://www.bv-brc.org/view/Genome/1218315.22

^2^ https://ggdc.dsmz.de/ggdc.php

^3^ https://pubmlst.org/organisms/brucella-spp

^4^ https://www.cgmlst.org/ncs
