# Supplementary material for: Isolation of Brucella inopinata from a White’s tree frog (Litoria caerulea): pose exotic frogs a potential risk to human health?
Source: Front Microbiol. 2023 Jun 8;14:1173252. doi: 10.3389/fmicb.2023.1173252 (PMC10285381; doi:10.3389/fmicb.2023.1173252)
Supplement: Supplementary file 5 [file Table_5.docx]

**Supplementary Table 5 | BLAST results with query coverage in top plasmid hit.**

| **Region** | **Contig** | **Start** | **Stop** | **Size** | **Query Coverage** | **Plasmid genome** |
| --- | --- | --- | --- | --- | --- | --- |
| One | Contig 1 | 225617 | 231305 | 5688 |  |  |
| Two | Contig 1 | 482167 | 500001 | 17834 |  |  |
| Three | Contig 1 | 791584 | 795002 | 3418 |  |  |
| Four | Contig 1 | 1017537 | 1021124 | 3587 |  |  |
| Five | Contig 1 | 1366970 | 1370851 | 3881 |  |  |
| Six | Contig 1 | 1381494 | 1394602 | 13108 |  |  |
| Seven | Contig 1 | 1545838 | 1570098 | 24260 |  |  |
| Eight | Contig 2 | 65238 | 72094 | 6956 | 42% | *Phyllobacterium* sp. Tri-48 strain |
| Nine | Contig 2 | 157992 | 168787 | 10795 | 39% | *Ochrobactrum anthopi* OAB |
| Ten | Contig 2 | 601007 | 628634 | 27627 |  |  |
| Eleven | Contig 2 | 691665 | 693308 | 1643 |  |  |
| Twelve | Contig 2 | 725202 | 728981 | 439946 |  |  |
| Thirteen | Contig 2 | 1022453 | 1053590 | 31137 |  |  |
| Fourteen | Contig 2 | 1053592 | 1088122 | 34530 |  |  |
| Fifteen | Contig 2 | 1113198 | 1131328 | 18130 |  |  |
| Sixteen | Contig 2 | 1147047 | 1178457 | 31410 | 15% | *Rhizobium leguminosarum* bv. Trifoli WSM1325 |
| Seventeen | Contig 2 | 1214615 | 1223889 | 885501 | 12% | *Rhizobium etli* NXC12 |
