# Supplementary figures and images for: Isolation of Brucella inopinata from a White’s tree frog (Litoria caerulea): pose exotic frogs a potential risk to human health?
Source: Front Microbiol. 2023 Jun 8;14:1173252. doi: 10.3389/fmicb.2023.1173252 (PMC10285381; doi:10.3389/fmicb.2023.1173252)

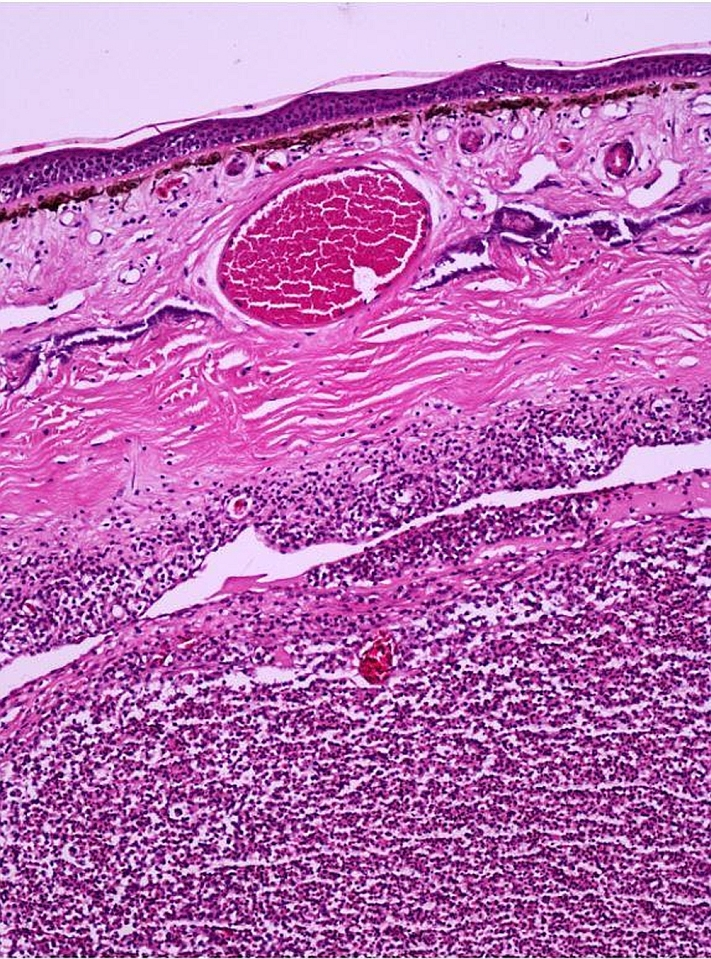

Supplement: Supplementary file 6 [file Image_1.TIF]

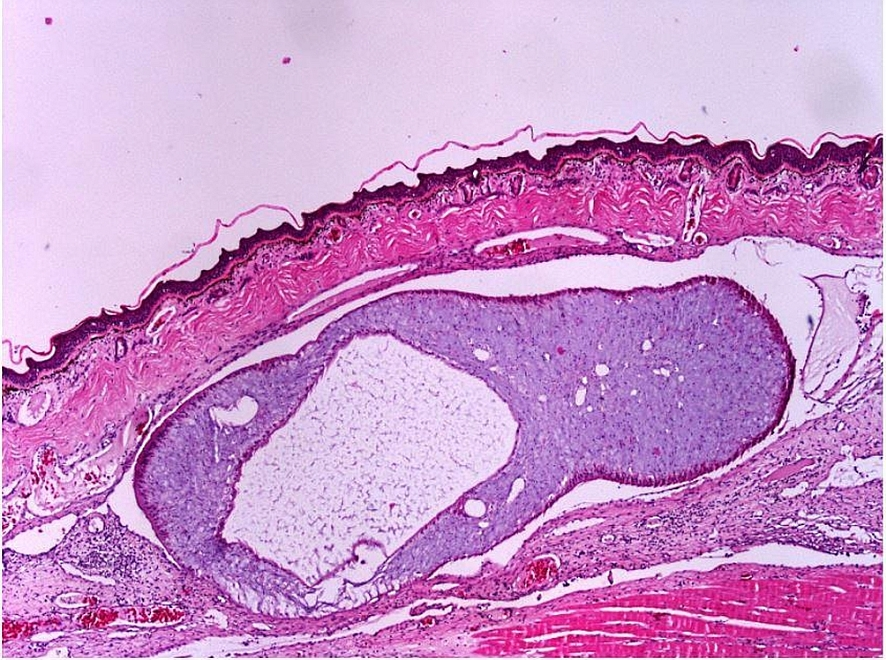

Supplement: Supplementary file 7 [file Image_2.TIF]

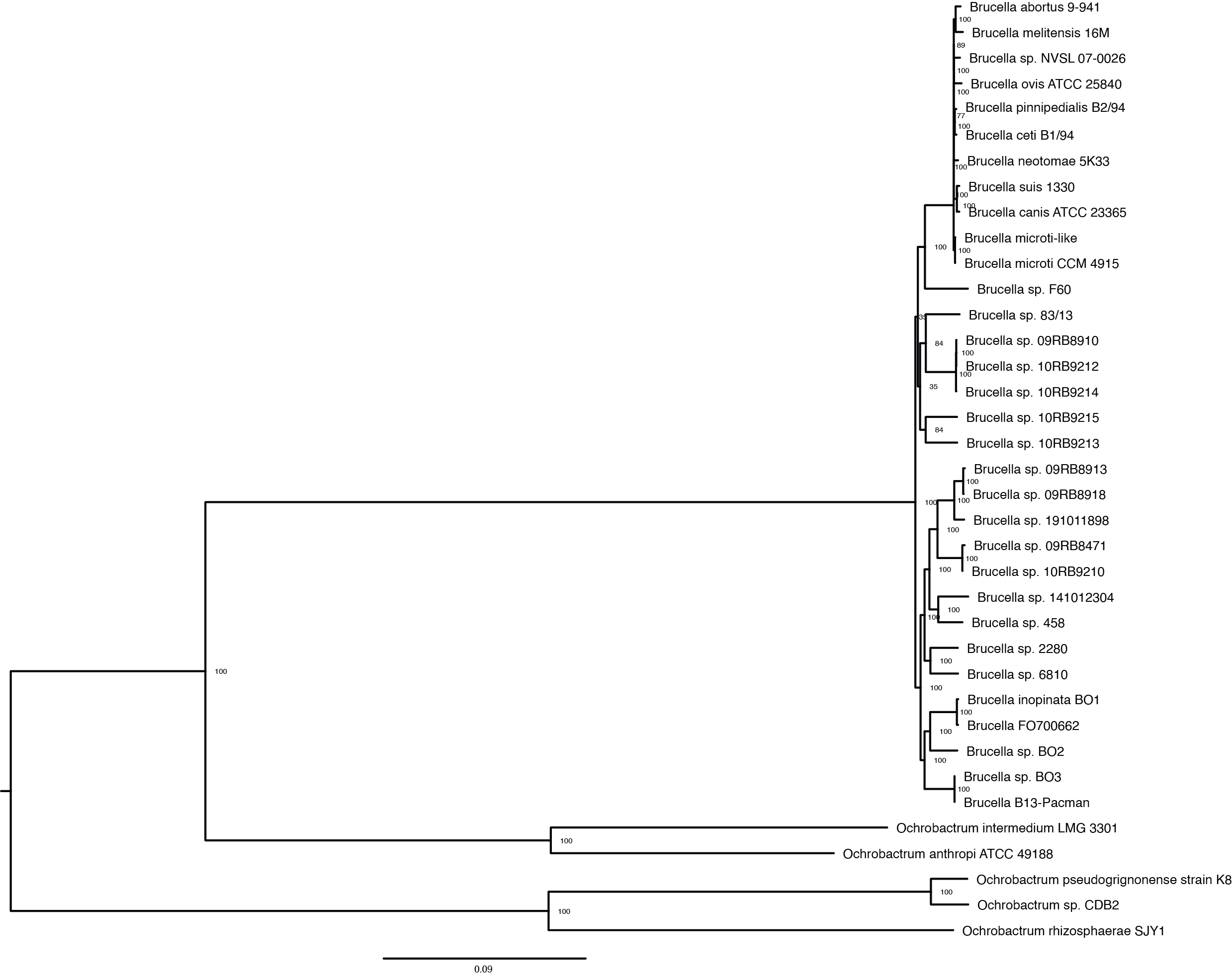

Supplement: Supplementary file 8 [file Image_3.TIF]

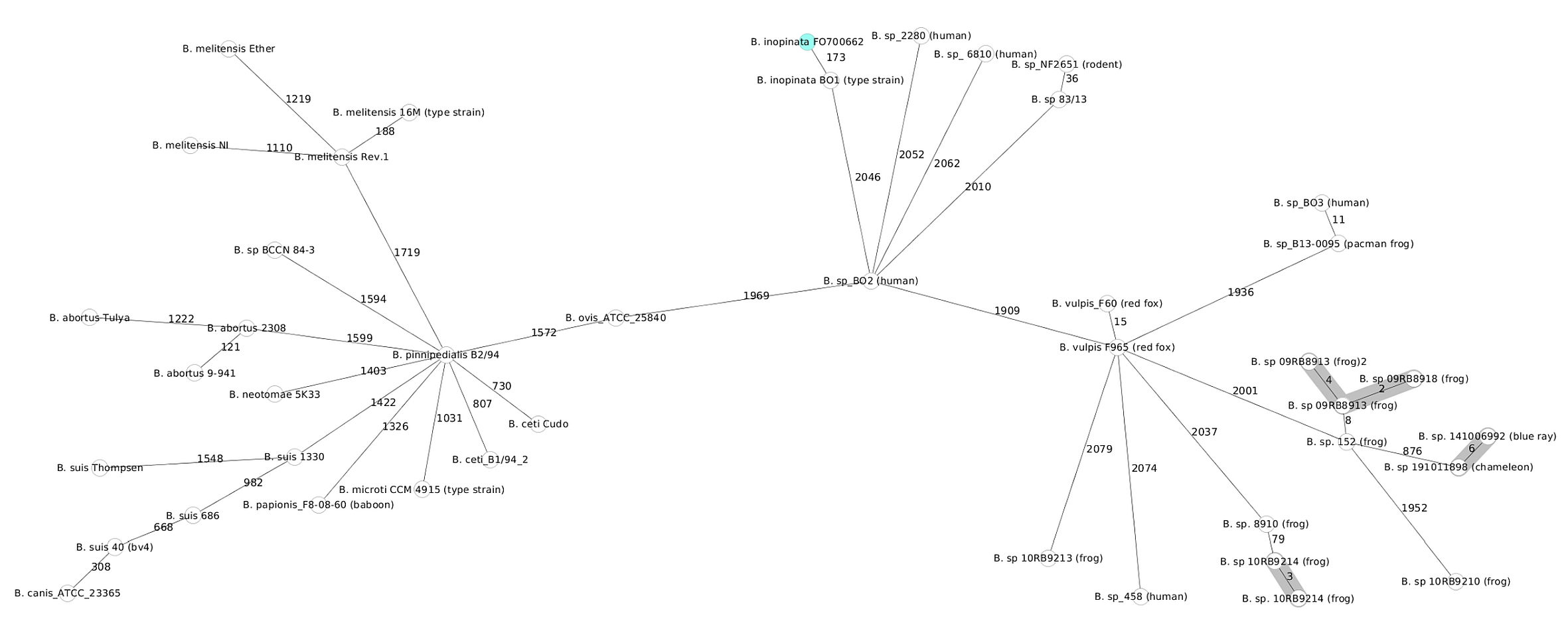

Supplement: Supplementary file 9 [file Image_4.TIF]
